# *Arabidopsis thaliana:* A Model Host Plant to Study Plant–Pathogen Interaction Using Rice False Smut Isolates of *Ustilaginoidea virens*

**DOI:** 10.3389/fpls.2016.00192

**Published:** 2016-02-23

**Authors:** Mebeaselassie Andargie, Jianxiong Li

**Affiliations:** Key Laboratory of South China Agricultural Plant Molecular Analysis and Genetic Improvement, and Guangdong Provincial Key Laboratory of Applied Botany, South China Botanical Garden, Chinese Academy of SciencesGuangzhou, China

**Keywords:** *Arabidopsis thaliana*, endophytic colonization, plant–pathogen interaction, plant defensin gene, PR genes, *Ustilaginoidea virens*

## Abstract

Rice false smut fungus which is a biotrophic fungal pathogen causes an important rice disease and brings a severe damage where rice is cultivated. We established a new fungal-plant pathosystem where *Ustilaginoidea virens* was able to interact compatibly with the model plant *Arabidopsis thaliana*. Disease symptoms were apparent on the leaves of the plants after 6 days of post inoculation in the form of chlorosis. Cytological studies showed that *U. virens* caused a heavy infestation inside the cells of the chlorotic tissues. Development and colonization of aerial mycelia in association with floral organ, particularly on anther and stigma of the flowers after 3 weeks of post inoculation was evident which finally caused infection on the developing seeds and pod tissues. The fungus adopts a uniquely biotrophic infection strategy in roots and spreads without causing a loss of host cell viability. We have also demonstrated that *U. virens* isolates infect *Arabidopsis* and the plant subsequently activates different defense response mechanisms which are witnessed by the expression of pathogenesis-related genes, *PR-1, PR-2, PR-5, PDF1.1*, and *PDF1.2*. The established *A. thaliana*–*U. virens* pathosystem will now permit various follow-up molecular genetics and gene expression experiments to be performed to identify the defense signals and responses that restrict fungal hyphae colonization in planta and also provide initial evidence for tissue-adapted fungal infection strategies.

## Introduction

Rice (*Oryza sativa*) false smut disease, caused by the the pathogenic ascomycete fungus *Ustilaginoidea virens* (Cooke) Tak (teleomorph: *Villosiclava virens*) is one of the most severe and devastating diseases of rice, which feeds one-half of the world’s population (Ford et al., 1994; Talbot and Foster, 2001). Since the hyphae extend into the central vascular tissues, this fungus is considered to be a biotrophic parasite (Tang et al., 2013). The infection process of *U. virens* was recently investigated through extensive histological and microscopic examinations (Tang et al., 2013; Wenlu et al., 2013; Mebeaselassie et al., 2015a). *U. virens* hyphae enter primarily at the upper parts of the floral organs through the palea and lemma in order to colonize the inner part of the spikelet. At a later stage of *U. virens* colonization, the basal area of the floral region witnessed the presence of a dense mass of mycelia due to the conducive micro-environment in terms of nutrients and moisture content. The *U. virens* occasionally infects the anther, stigma, ovary and lodicules (Mebeaselassie et al., 2015a). A 39.4Mb draft of *U. virens* genome that encodes 8,426 predicted genes has been sequenced (Zhang et al., 2014). In addition to this, the pathogen has showed a decreased gene inventories for different metabolisms including nutrient uptake and polysaccharide degradation. This could arise possibly due to the adaptation of the pathogen to the specific floret infection and biotrophic lifestyles.

There are different possibilities that can be observed when a plant is attacked by a pathogen. The pathogen can proliferate and grow fastly on the plant at a rate in which the plant could not be able to control the fast growth of the pathogen which subsequently leads to the development of disease and necrosis. There is also a possibility for the plant to resist the invasion and colonization induced by the pathogen and resistance could be either active or passive. The presence of a resistance gene is responsible to condition an active resistance in plants, whose product directly or indirectly recognizes a race-specific avirulence determinant produced by the pathogen (Keen, 1990; Scofield et al., 1996; Tang et al., 1996). One of the most visible signs that a plant is resisting pathogen attack is the development of a hypersensitive response (HR) on the inoculated tissue. The HR is characterized by small necrotic lesions that form around the infection site (Matthews, 1991). These lesions help restrict the growth and spread of the pathogen (Slusarenko et al., 1991). Similarly, expressions of pathogenesis-related (PR) proteins as well as plant defensin 1.1 and 1.2 (PDF1.1, PDF1.2) mRNAs are some of the responses that are mediated by gene-for-gene interactions in addition to the rapid localized cell death (Narasimhan et al., 2001; Asano et al., 2012). Jasmonic acid (JA), Salicylic acid (SA), ethylene (ET), and reactive oxygen species (ROS) are some of the signaling molecules that are directly entailed in the above mentioned inducible defense systems (Clarke et al., 2000; Kunkel and Brooks, 2002; Hossain et al., 2007; Asano et al., 2012).

Due to the presence of different defense mechanisms displayed by the plants, different species of plants are somehow susceptible to only a limited number of pathogens even though there are large number of disease causing agents found in nature (Nimchuk et al., 2003; Jones and Takemoto, 2004). Plant disease resistance, molecular and cellular basis of host–pathogen interactions and pathogen virulence studies using different model systems were previously reported by Bohman et al. (2004), O’Connell et al. (2004), Park et al. (2009).

The availability of complete genome sequence and having a small genome size together with the extensive collection of new mutants and germplasm as well as the presence of specialized transformation techniques made *Arabidopsis* (*Arabidopsis thaliana*) to serve as a good genetic and molecular model for plant biology research in general and for plant–pathogen interaction studies in particular. In addition to this, it has a rapid growth, can be handled easily in the laboratory condition and mutagenesis can be done easily. *Arabidopsis* is susceptible to only a limited number of pathogens including viruses, bacteria, fungi, nematodes and insect pests and it responds to the pathogen attack in a similar fashion to those of other higher plant species. Though, *A. thaliana* has been used extensively as a model plant to make clear the plant–pathogen interactions with a wide variety of pathogens since the 1990s, there is no report showing the infection of *Arabidopsis* by *U. virens* pathogens to date.

Generally, for a pathogen to be successful, it should be able to evade host defense then colonize host tissues or organs and propagate within the host. Defense is mediated either through the SA-dependent or the JA/ET-dependent pathway against biotrophic and necrotrophic pathogens, respectively (Delaney et al., 1994; Thomma et al., 1998). Signaling pathways controlled by SA, JA, and/or ET are involved in controlling the interaction of *Arabidopsis* and rice with different pathogens. *Arabidopsis* NPR1 protein is an important regulatory component in plant immunity, controlling the onset of systemic acquired resistance (SAR) however, infection of rice spikelets by *U. virens* suppressed expression of rice defense-related genes homologous to *NPR1, PR1*, CNGC, and *AtMIN7* in *Arabidopsis* (Fan et al., 2015) which suggests that the SAR pathway in rice spikelets may be suppressed upon *U. virens* infection. Rice and *Arabidopsis* have a slightly different disease phenotype, i.e., *U. virens* infection converts individual grains into smut balls, which results in sterility of the florets in rice while smut ball formation is not observed in *Arabidopsis* florets though sterility of the flowers is evident; however, they share similar defense response mechanisms. Proteins that are involved in protein modification, protein degradation and receptor phosphorylation were greatly activated during the first stage of *U. virens* infection in the infected spikelet. During this time, some receptor protein kinases can activate corresponding substrates to facilitate downstream signal transduction and this is shown by MPK3 and MPK6, which phosphorylate WRKY33 to initiate phytoalexin biosynthesis in *Arabidopsis* (Chao et al., 2014). Important biological processes that have a great role for the plant development like ion transport, carbohydrate and cellular lipid metabolic process were repressed as the disease progresses which suggests that infection by *U. virens* pathogen inhibits the normal growth and development of plant floral organs. In addition, a protein kinase APK1B which is involved in stamen development and its repression can prevent pollen tube germination causing self incompatibility in *Arabidopsis* was observed at a later stage of *U. virens* rice spikelet infection suggesting that the pathogen manipulates host development signaling by prohibiting protein phosphorylation, hence allowing further infection of the plant with *U. virens* to occur (Chao et al., 2014).

The use of the GFP reporter gene as well as the GUS gene fusion system provided valuable information about different plant–pathogen systems and has been used for a number of live-imaging applications with plant pathogens (Couteaudier et al., 1993; Lagopodi et al., 2002; Kankanala et al., 2007; Hu et al., 2014; Mebeaselassie et al., 2015a).

In rice, definitive plant molecular genetics experiments in relation to *U. virens* infection are difficult to conduct because of unavailability of suitable resistant germplasm sources for this fungal disease and in addition it also needs long generation times. We therefore decided to explore whether *A. thaliana* could be exploited for high-throughput molecular genetic studies by establishing a new model pathosystem based on *Arabidopsis* as the host and *U. virens* as the pathogen. In this paper, we describe experiments that show the rice fungal pathogen, *U. virens* colonize and attack the leaves, flowers, pods, and roots of a dicotyledonous plant, *A. thaliana.* Through gene expression analysis, we also confirmed that different defense response proteins were induced in the infected *Arabidopsis* plants. This compatible model system will now permit various follow-up molecular genetics and gene expression experiments to be performed to identify the defense signals and responses that restrict fungal hyphae colonization *in planta*.

## Materials and Methods

### Plant Material and Growth Conditions

The wild-type seeds of *A. thaliana* ecotype Columbia were sown on wet mixture of soil and vermiculite in plastic pots. Seeds were surface sterilized in 0.1% Triton X-100 for 30 min, 0.1% Triton X-100 plus 70% ethanol for 5 min and 0.1% Triton X-100 plus 2% commercial bleach for 10 min, washed three times in sterile distilled water, and placed in the petri dishes containing Murashige and Skoog agar (0.8%, w/v) medium. Plants were grown under a 16/8 h light/dark cycle photoperiod at 22°C.

### Construction of a GFP and GUS-Containing Strain of *U. virens*

Several GUS-tagged strains of *U. virens* were obtained by transforming fungal spores with plasmid pCAMBIA1301, which contains the *Escherichia coli* β-glucuronidase gene (*gus*A) flanked by a glyceraldehyde 3-phosphate promoter (*gpd*) from *Aspergillus nidulans* upstream and the *A. nidulans trp*C transcription termination signal downstream, whereas pBHt1- sGFP which was used to generate GFP-tagged strains of *U. virens* was a kind gift from Prof. Hua-Ping Li, South China Agricultural University. *Agrobacterium* mediated transformation was performed as described by Mebeaselassie et al. (2015a), selection for transformants was done using PSA amended with 100 mg/liter hygromycin B (PSA-hyg).

### Plant Inoculation and Fungal Growth

A single spore culture of the transformed *U. virens* was seeded into a potato sucrose medium and incubated for 7 days as previously described (Mebeaselassie et al., 2015a). The conidial suspension was adjusted to 5 × 10^5^ conidia mL^-1^ with sterile distilled water and used as the inoculum. Ten 4-week-old *Arabidopsis* plants were sprayed with 20 mL of the conidial suspension using an air brush. Upon infection, in order to increase the relative humidity the plants were kept inside plastic bags. Each inoculation experiment was repeated three times. Mock inoculation was done by spraying 20 mL of sterile distilled water for spores. For root infection, 3-week-old *Arabidopsis* seedlings were inoculated with conidia of *U. virens* by unimpaired root dip-inoculation. Seedlings were aseptically transferred into tubes containing a sterile nutrient solution. The plants were cultivated in a growth chamber at 22°C. Control plants were dipped in sterile distilled water and then cultivated, as were the inoculated plants. Radicles were sampled after 24 hpi (hours post- inoculation) and a close observation of the different parts of the roots continued for 4 weeks.

### Analysis of GFP Fluorescence

Microscopic examinations of leaves were carried out at multiple times (1, 2, 3, 4, 5, 6, and 7 days) whereas for flowers and pods examinations were carried out after 3 weeks of inoculation. Systemic colonization by *U. virens* in *Arabidopsis* root tissues was determined at 1, 2, 3, 4, 5, 10, 15, 18, 21, 24, 27 days and 4 weeks post-inoculation (dpi). A minimum of 10–20 roots were analyzed for each time point. The roots were divided into 1–1.5 cm sections before they were mounted on a glass slide. Generally, all objects were placed on a glass slide in a water droplet, covered with a cover slip, and observed without further manipulation. Microscopic observations were carried out using a LeicaDM6000 B microscope with excitation at 455–490 nm and emission at 515–560 nm at 40 and 100× magnification.

### Histochemical Localization of GUS Activity

Different parts of the *Arabidopsis* plant were evaluated for GUS activity after 12 h incubation at 37°C with the substrate 5-bromo-4-chloro-3-indolyl glucuronide, cyclohexylammonium salt (X-gluc, Biosynth AG, Staad, Switzerland), as described previously by Couteaudier et al. (1993). After staining, tissues were cleared by replacing the staining solution with several changes of 70 and 90% (v/v) ethanol as deemed necessary and it was viewed under a stereomicroscope. For *Arabidopsis* root observation, semi-thin sectioning was done as it was described by Wenlu et al. (2013) and colonization of the fungus inside the root was viewed under a stereomicroscope.

### RNA Isolation and RT-PCR Analysis

Total RNA was isolated from 0.15 g of leaf and root materials at 0, 24, 48, 72, and 96 dpi and also from 5-week-old flowers and pods of *A. thaliana* that were inoculated and infected with *U. virens* according to the method described by Meisel et al. (2005) using the plant RNA extraction kit (Applygen Technologies Inc., Beijing, China). The concentration of isolated total RNA was calculated from absorbance at 260 nm with a ND-1000 spectrophotometer (NanoDrop, Wilmington, DE, USA), the quality was verified by optical density (OD) absorption ratio OD_260__nm_/OD_280__nm_ between 1.80 and 2.11, and OD_260__nm_/OD_230__nm_ ranging from 2.00 to 2.48 and the integrity was evaluated by electrophoresis on ethidium bromide-stained 1.0% agarose/TAE gels. Finally the cDNA was synthesized using the PrimeScript RT kit (Takara Bio, Shiga, Japan) reagent. The qRT-PCR was carried out with SYBR^®^Premix Ex Taq^TM^II (Perfect Real Time; Takara Bio, Shiga, Japan) together with gene specific primer pairs for *PR1* (5′-GTAGGTGCTCTTGTTCTTCC-3′; 5′-TTCACATAATTCCCACGAGG-3′), *PR2* (5′-TCAAGGAAGGTTCAGGGATG-3′; 5′-TCGGTGATCCATTCTTCACA-3′), *PR5* (5′-ATGGCAAATATCTCCAGTATTCACA-3′; 5′-ATGTCGGGGCAAGCCGCGTTGAGG-3′), *PDF1.1* (5′-GAGAGAAAGCTTGTTGTGCGAGAGGCCAAGTGGG-3′; 5′-GAGAGAGGATCCTGCAAGATCCATGTCGTGCTTTC-3′) and *PDF1.2* (5′- AATGAGCTCTCATGGCTAAGTTTGCTTCC-3′; 5′- AATCCATGGAATACACACGATTTAGCACC-3′) and *ACTIN* (5′-GCACCCTGTTCTTCTTACCG-3′; 5′-AACCCTCGTAGATTGGCACA-3′), respectively, and cDNA as template. The PCR cycle conditions were as follows: 95°C, 10 s; 40 cycles of 95°C for 5 s, 60°C for 20 s, and 72°C for 30 s. Data were normalized to the reference gene Actin, and the transcript level relative to the control materials was determined for each sample using the delta–delta CT (ΔΔCT) method, where ΔΔCT *=* (*C*_T_, _Target_–*C*_T_, _Actin_) _Time_ *_x_*–(*C*_T_, _Target_–*C*_T_, _Actin_) _Time0_ (Livak and Schmittgen, 2001). At first, the threshold cycles (CT) of the duplicate PCR results of each gene were averaged and used for quantification of the transcripts. Then, the average of the CT value of the actin gene was subtracted from the average of the CT value of the target gene to obtain the ΔCT value. The 2^-ΔΔ^CT value was given to estimate the relative expression rate of each gene. Each value was obtained from three independent experiments. A standard deviation was given to each value and the results were analyzed by the Student’s *t*-test.

## Results

### Microscopy of Pathogen Development in *A*. *thaliana* Leaves

**FIGURE 1:**
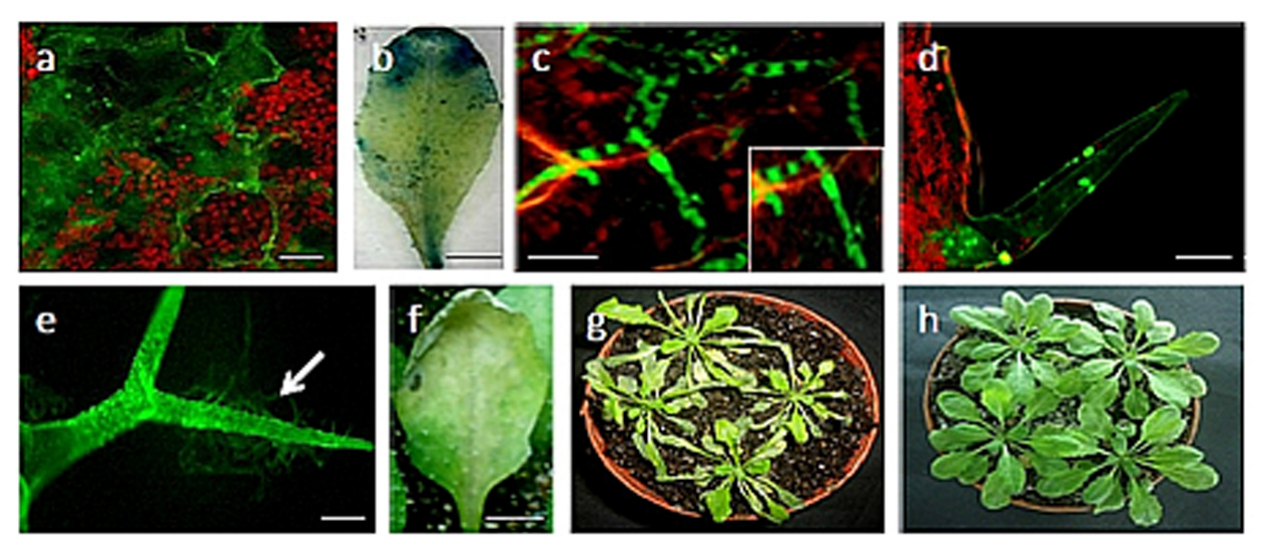
**Infection of 4-week-old *Arabidopsis thaliana* plants (ecotype Columbia) with *Ustilaginoidea virens*. (a)** Germinating conidia on the surface of the leaf; **(b)** GUS stained *Arabidopsis* leaf after 72 hpi; **(c)** a branched mycelium proliferating on the leaf after 72 hpi. The inset shows magnification of the section; **(d)** and **(e)** spores and hypha attached on trichomes; **(f)** formation of chlorotic lesion at the site of inoculation of *U. virens* isolates after 96 hpi; **(g)** Infected *Arabidopsis* plants after 3 dpi; **(h)** Control *Arabidopsis* plants. Bars = 100 μm **(a,d,e)**, 20 μm **(c)** and 50 μm **(b,f)**.

**FIGURE 2:**
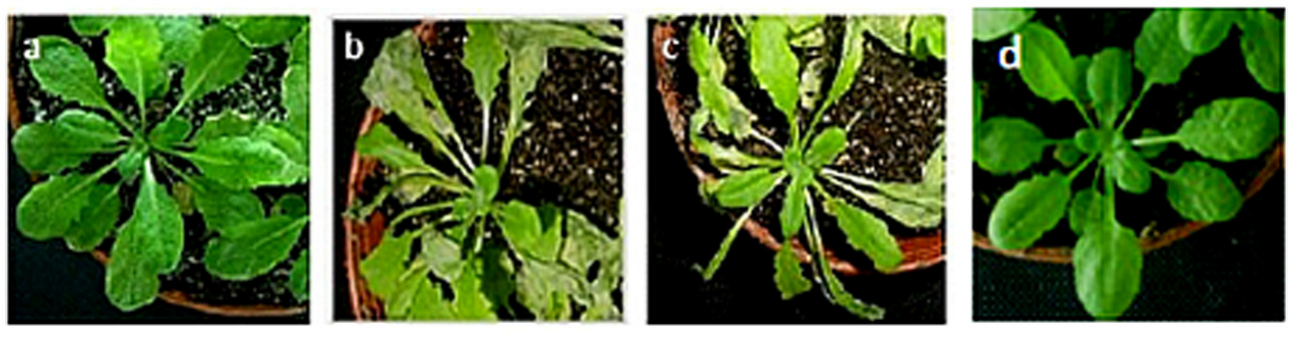
**Progression of disease symptoms caused by *U. virens* infection.** The disease symptoms in *Arabidopsis* ecotype, Col-0: **(a)** 0 dpi; **(b)** 3 dpi; **(c)** 6 dpi; **(d)** Control.

The transformed *U. virens* isolate tagged with both GFP and GUS marker genes was easily detected in infected *Arabidopsis* leaf tissues (**Figures 1a,b**). Conidia were able to attach and germinate on the surface of the leaves several hours after inoculation and at 48 hpi where the biotrophic hyphae can only exist, a large amount of mycelium with many branches developed on the surface of the leaf (**Figures 1c** and **2c**). The GFP and GUS labeled fungus infected almost all parts of the leaf and it was also observed on leaf trichomes (**Figures 1d,e**). The mycelium covered the inoculated tissues 3 days post inoculation and it has no special order of growth on the surface of the leaves (**Figure 1c**). The responses of Columbia (Col-0) to *U. virens* illustrate the nature and progression of disease symptoms (**Figures 1f,g** and **2c**). Plants of Col-0, which were exposed to *U. virens*, began producing small chlorotic or yellow spots within 3 days of post-inoculation (dpi; **Figures 1h** and **2b**). Chlorotic lesions were also evident after 96 hpi at the different parts of the infected leaf. Whereas no symptom was observed on the uninfected and infected Col-0 after 0 dpi (**Figures 2a,d**). These spots subsequently expanded and became highly visible by 6 dpi (**Figures 1f,g**).

### Microscopy Studies of *U. virens-Arabidopsis* Flower Interaction

**FIGURE 3:**
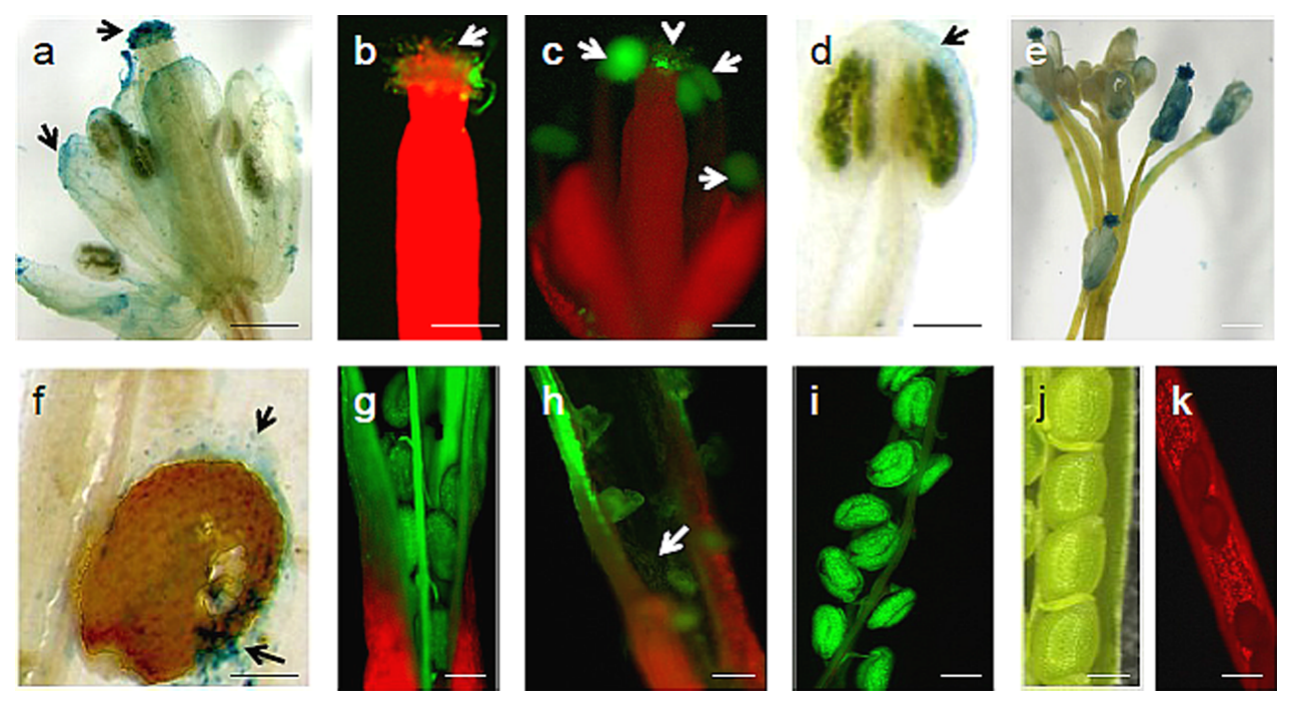
***U. virens*-*Arabidopsis* flower interaction. (a)** Visible intense GUS histochemical staining on different floral regions of infected *Arabidopsis* flower by *U. virens* 3 weeks after post-inoculation; **(b)** GFP tagged *U. virens* hyphae growing on the stigma 3 weeks after post-inoculation; **(c)** GFP-tagged aerial mycelia on the male and female parts of *Arabidopsis* flower 3 weeks after post-inoculation; **(d)** *U. virens* infected male floral structure showing the GUS stain; **(e)** Colonization of GUS labeled aerial mycelia on the stem, anther, and stigma of the flowers after 3 weeks of post inoculation; **(f–i)** Mycelium development on siliques and seeds as well as shriveled pod formation from infection of flower tissue 28 days after inoculation with GFP and GUS labeled and transformed strains of *U. virens*; **(j)** Control showing *Arabidopsis* siliques and seeds; **(k)** Uninfected *Arabidopsis* pod under epifluorescence microscopy. Bars = 40 μm **(g–j)**, 20 μm **(a–d,f,k)**, 150 μm **(e)**.

Spray inoculation of conidia onto the leaves of *Arabidopsis* ecotype Columbia-0 resulted in the development and colonization of aerial mycelia in association with anther and stigma of the flowers after 3 weeks of post inoculation (**Figures 3a–c**). Subsequently, *Arabidopsis* flowers showed the visible intense fluorescence and the GUS histochemical staining. Colonization proceeded into the petals, stigma, anther, and peduncle tissues shortly (**Figures 3a–e**). The pods of the infected *Arabidopsis* flower after 4 weeks of post inoculation had shriveled and dried; lastly it turned brown. Microscopic observations revealed hypha colonization of the mature as well as the immature seeds and the inner layers of the pod (**Figures 3f–h**). The intense colonization which was observed on the floral organ after *U. virens* infection makes the developing seeds and pod tissue to become infected strongly (**Figure 3i**). However, no green fluorescence was observed in the pod of the uninfected *Arabidopsis* plants (**Figures 3j,k**).

### Endophytic Development in *Arabidopsis* Roots

**FIGURE 4:**
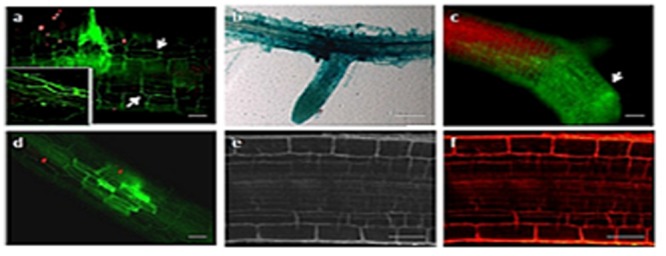
**Early colonization stages of *Arabidopsis* roots by *U. virens*. (a)** hyphae growing along the epidermis parallel to the longitudinal axis of the root and forming lateral hyphopodia (arrow), 2 dpi. The inset shows magnification of the upper section of the root; **(b)** younger GUS stained hyphae extensively colonize the root surface, forming a network around the root; **(c)** heavy colonization of the *Arabidopsis* root by *U. virens* after 10 dpi; **(d)** root cells become colonized by hyphae in a mosaic pattern, leaving some cells uninfected after 10 dpi; **(e)** root of the uninfected *Arabidopsis* plant under bright field microscopy (Control); **(f)** uninfected *Arabidopsis* root under epifluorescence microscopy. Bars = 100 μm **(a,c,d)**, 40 μm **(e,f)** and 50 μm **(b)**.

**FIGURE 5:**
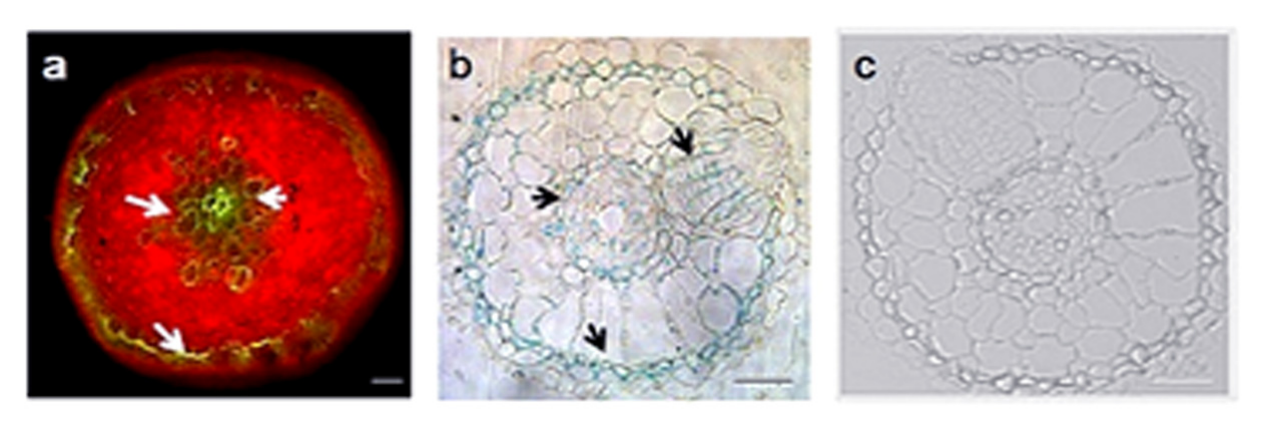
**Advanced colonization stages of *Arabidopsis* root by *U. virens* expressing GFP and GUS histochemical staining. (a)** cortical cells are colonized by GFP-tagged hyphae (*arrow*) after 21 dpi; **(b)** semi-thin sectioning of *Arabidopsis* root showing a heavy colonization of epidermis, cortex and the endodermis after 21 dpi by the GUS labeled hyphae; **(c)** Semi-thin sectioning of an uninfected 21-day-old *Arabidopsis* root under bright field microscopy (Control). Bars = 100 μm **(a)** and 20 μm **(b,c)**.

To characterize *U. virens* infection of *Arabidopsis* roots, we inoculated roots of *in vitro* cultivated *Arabidopsis* plants with conidia of GFP as well as GUS expressing *U. virens* and documented infection over a period of 3 weeks by fluorescence microscopy and GUS histochemical staining (**Figures 4a–d**). Colonization initiates from conidia, which, upon germination, finally form a hyphal network on and inside the root. After the conidia germinated and producing fungal hyphae, numerous runner hyphae were observed along the longitudinal axis of the root surface after the second day of post inoculation (**Figure 4a**). From 3 to 4 dpi, hyphae continued to colonize the root surface growing along the junctions of the epidermal cells and outlining the cell borders forming a fluorescent pattern. In most cases, the hyphae growth followed a long route along the longitudinal axis (**Figure 4a**). Basically, fungal hyphae entered the tissue mainly through rhizodermal cells since there was no evidence of penetration of root hairs observed. Fungal progression was characterized by successive invasions of rhizodermal cells with no apparent loss of cell viability. The hyphae appeared to be confined to the infected cell until the intracellular space was completely filled with hyphae. Only certain epidermal and cortical cells appeared to become colonized in this manner, while the surrounding cells remained uninfected, resulting in a mosaic pattern of plant cell colonization (**Figure 4d**). At 10 DPI a more complex hyphal networks that form a net-like structure along the root epidermis grow within the grooves between epidermal cells (**Figures 4b,c**) however, there was no fungal hypha and mycelia on the uninfected *Arabidopsis* root (**Figures 4e,f**). At later colonization stages, fungal hyphae had spread to inner cell layers of the root, including the cortex, the endodermis, and the vascular tissue (**Figures 5a,b**). The pathogen reached the cortex after invasion of the epidermis, and cortical cells were essential for successful colonization of the pathogen. Generally, *U. virens* exhibited both inter and intracellular invasion of *Arabidopsis* root cell layers and rapidly colonized the root, including the vascular tissue, without causing necrosis or showing no microscopic cell death within the assessed period of up to 3 weeks of post infection. No fungal structure was observed in the cross-section of the uninfected *Arabidopsis* root (**Figure 5c**).

### Induction of Pathogenesis Related Genes in Response to *U. virens* Infection

**FIGURE 6:**
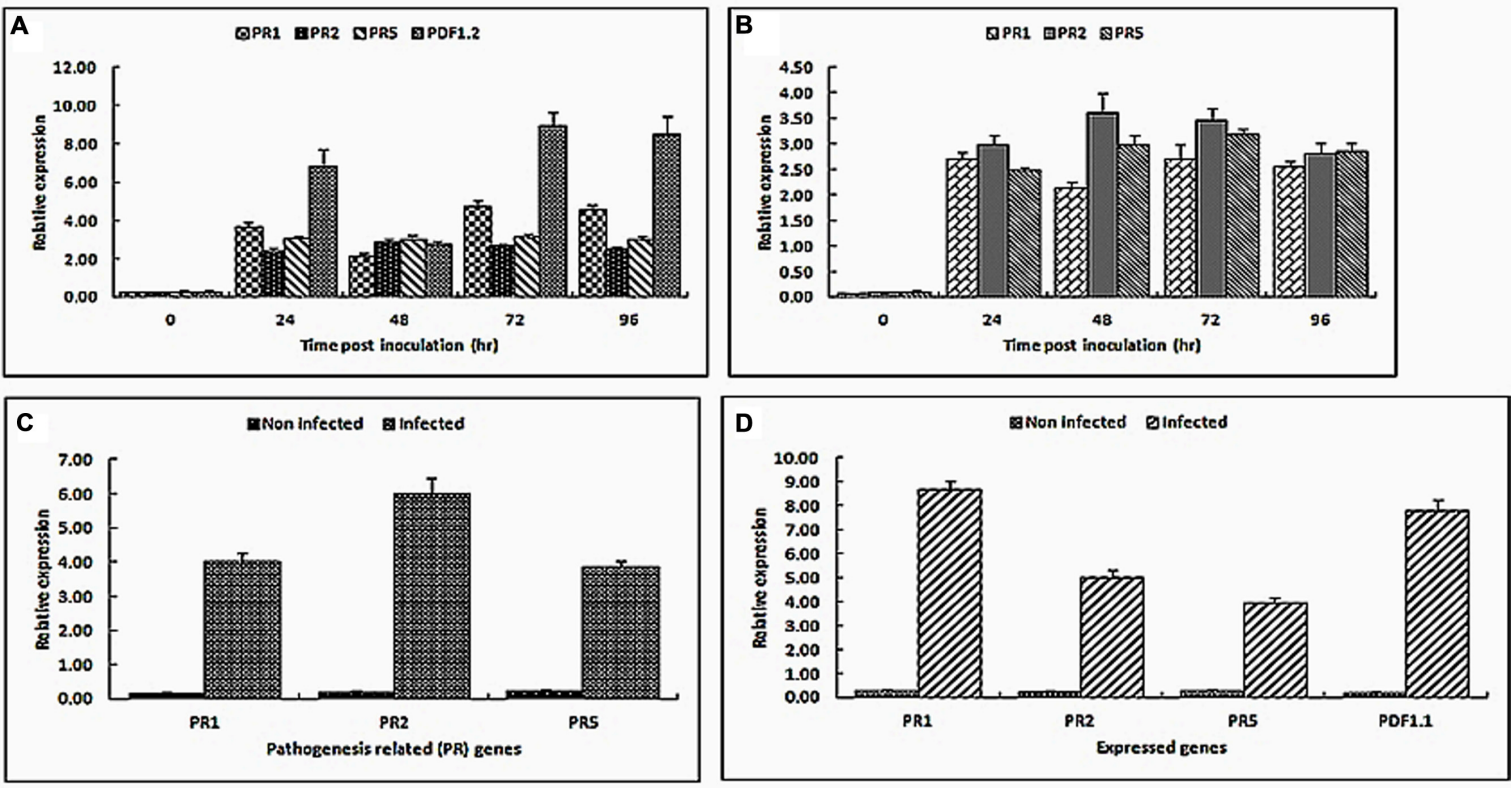
**Real-time RT-PCR analysis of *PR1, PR2, PR5, PDF1.1*, and *PDF1.2* gene expression in *Arabidopsis* leaves **(A)**, roots **(B)**, flowers **(C)**, and pods **(D)** upon a treatment with *U. virens*.** *Arabidopsis* leaves and roots were inoculated by adding a suspension of germinated microconidia in order to reach the concentration of 5 × 10^5^ conidia mL^-1^. Control leaves, flowers, roots, and pods were treated with sterile distilled water. Expression levels were normalized with respect to the housekeeping gene *Actin*. Data bars represent the mean ± SD of three repeats of the transcripts assessed at 0, 24, 48, 72, and 96 h post-inoculation for leaves and roots while flowers and pod gene transcripts were assessed 5 weeks post inoculation.

Taking the Actin as a housekeeping gene, semi-quantitative RT-PCR analysis was performed with the RNA isolated from leaves, roots, flowers, and pods of *Arabidopsis* plants following infection with the *U. virens* spores in order to test whether the pathogenesis related genes as well as plant defensin genes were inducible by pathogen infection (**Figure 6**).

The leaf samples for analysis were collected at 0, 24, 48, 72, and 96 hpi. During the first 24 hpi, the expression of all *PR1, PR2, PR5*, and *PDF1.2* were strongly induced at 24 h then it was relatively weakly induced at 48 hpi, but at 72 hpi and there after the expression level of *PR1* and *PDF1.2* gradually increased and induced strongly again. Obviously, the expression level of *PDF1.2* and *PR1* genes was highest at 72 hpi (**Figure 6A**). Compared with mock-inoculated leaves, *PDF1.2* and *PR1* gene expressions were up-regulated greater than approximately eight and fourfold at 72 hpi. Both *PDF1.2* and *PR1* are established marker genes for the JA/ET mediated as well as SA signaling pathway, respectively. From this experiment, it has been shown that when *Arabidopsis* mounts a resistance response, the pathogenesis related genes were induced and they were related to the defense mechanisms against rice false smut.

To characterize *Arabidopsis* root invasion by *U. virens* at the molecular level, we used well-established foliar defense genes (*PR1, PR2*, and *PR5*). Gene expression analysis of all the three *PR* genes during root invasion revealed increased transcript accumulation in infected roots relative to the control roots (**Figure 6B**). Expression levels of *PR2* and *PR5* genes were highest at 2 and 3 DAI, followed by a reduction in expression levels at 4 DAI while levels of *PR1* transcript, by contrast, were significantly induced at the first and third days of post inoculation.

As shown in **Figures 6C,D**, *U. virens* infected *Arabidopsis* flowers and pods showed increased levels of *PR1, PR2*, and *PR5* as well as *PR1, PR2, PR5*, and *PDF1.1* transcripts, respectively, compared to the mock-inoculated flowers and pods. *PR1* gene was expressed greater than twofold in the pods than the floral organs whereas *PR2* and *PR5* genes were expressed almost equally in both the pods and the floral organs of the infected *Arabidopsis* plants. In addition to the *PR* genes, plant defensin gene, *PDF1.1* which is usually found in a very high amount in seeds and seedlings and initially speculated as having a primary role in protecting seeds and seedlings from soil-borne pathogens was expressed predominantly in the pods and seeds.

## Discussion

*Ustilaginoidea virens*, a pathogenic ascomycete fungus, infects rice and brings a devastating grain disease in the majority of rice-growing areas of the world and also produces large amounts of mycotoxins which frequently cause poisoning of animals. In this paper, we have demonstrated that *U. virens* isolates infect and colonize the different parts of *A. thaliana*. To our knowledge, colonization of *U. virens* on *Arabidopsis* has not been previously reported.

Fungal spores germinate on the leaves of *A. thaliana* within 24 h. Hypha is seen within 48 h and mycelium with 72 h. The heavy infection of *Arabidopsis* is related to the massive colonization of the intracellular spaces with large quantities of mycelium. The fast-developing mycelium colonizes the cells, causing chlorosis, and the fungus proliferates in almost three quarters of the infected plant leaves. It is thus concluded that the pathogen can colonize and proliferate on the surface of the inoculated organ by entering through the epidermal layers and the stomata. The fungus obtains nutrients from the plant apoplast spaces in sufficient amounts to keep it viable. By this mode of infection *U. virens* can survive on healthy looking plants for long periods of time as it was reported previously by Freeman et al. (2001) on other pathogens. Despite the germination and growth of these fungal spores, our results demonstrate that *A. thaliana* has activated a HR mechanism which precedes a slower systemic response that ultimately leads to SAR in order to limit or reduce the damage and destruction caused by this biotropic fungus. In addition, it was clearly observed microscopically that the progression of the fungal hyphae on the surface of the leaves through the attachment to the trichomes. Similar observations were reported by Chamberlain (1975) and Kunkel and Brooks (2002) in apple trees. So it seems that the feature of leaves not only influence the retention of fungal spores but also determines the survival, attachment, and penetration of the hyphae due to the presence of trichomes on the leaf surface. Trichomes facilitate the fungal infection by acting as a physical adhesion point for the hyphae in addition to retaining water on the plant surface and provide nutrients for microbial growth (Lindow and Brandl, 2003; Monier and Lindow, 2003; Calo et al., 2006). Therefore, in addition to the attachment of the spores, trichome structures may also provide a protected microenvironment for fungal growth.

The data presented in this study also clearly indicates that the fungal hyphae which arises from the air-borne fungal spores was able to enter via colonizing the anther and then the filament or to penetrate directly into the top of the exposed stigma as each flower opens in order to progress and colonize the whole floral tissue of *Arabidopsis* plant. These infections extend into the seed pods and cause damage of the seeds. The presence of extensive aerial mycelia that developed in association with anther and stigma tissue during the infection was a striking feature for *Arabidopsis* flowers. The infection spreads into other floral tissues in order to reach eventually the pods and developing seeds. In rice, *U. virens* infection converts infected rice grains into smut balls which results in sterility of the florets whereas in *Arabidopsis* sterility of the flowers is evident without smut ball formation. Basically both *Arabidopsis* and rice share similar defense response mechanisms. Chao et al. (2014) reported that during the first stage of *U. virens* infection in the infected rice spikelet, proteins that are involved in protein modification, protein degradation and receptor phosphorylation were greatly activated; during this time, some receptor protein kinases can activate corresponding substrates to facilitate downstream signal transduction and this is shown by MPK3 and MPK6, which phosphorylate WRKY33 to initiate phytoalexin biosynthesis in *Arabidopsis*. In addition, a protein kinase APK1B which is involved in stamen development and its repression can prevent pollen tube germination causing self incompatibility in *Arabidopsis* was observed at a later stage of *U. virens* rice spikelet infection suggesting that the pathogen manipulates host development signaling by prohibiting protein phosphorylation, hence allowing further infection of the plant with *U. virens* to occur. Similar to the current finding, Urban et al. (2002) previously reported that *Fusarium* infections were capable to disperse into other floral structures of *Arabidopsis* plant while the infections were always contained within the open flowers without spreading further in tobacco, tomato, and soybean plants. In relation to the seed transmission, our result showed that *U. virens* was able to colonize and cause seed infection via flowers. Previous report by Oliver et al. (2001) showed that seed infection by *Alternaria brassicicola* occurred through the flowers of *Cakile maritima*. The microscopic observation showed intensive fungal colonization on the surface of the seeds which could probably be favored by the release of carbohydrates at the surface of damaged seeds as proposed by Knox-Davies (1979). Our observations did not allow us to determine whether *U. virens* was limited to the seed coat or also present inside the endosperm and embryo. However, previous report by Singh and Mathur (2004) showed that biotrophic fungi are often located in the embryo. To our knowledge, this is the first microscopic analysis that has ever been reported concerning pod and seed colonization by *U. virens* on *A. thaliana*.

The *U. virens* isolate entered through the root epidermis then colonize in the epidermal and cortical tissues. Previously, similar behavior of hyphae for other root endophytes has been reported by Gao and Mendgen (2006) and Zvirin et al. (2010). The fungi were able to colonize the epidermal layer intra and inter-cellularly and also able to reach to the cortex region which is a typical feature of the non-pathogenic root colonizers (Schardl et al., 2004). In addition, the early stages of *Arabidopsis* root infection by *U. virens* were characterized by extensive intercellular colonization and a mosaic pattern of intracellular colonization. This type of mosaic pattern of root colonization has been described for the different pathogenic interactions on different plant roots (Lagopodi et al., 2002; Vierheilig et al., 2005; Mebeaselassie et al., 2015b) which indicate that this pattern of infection is not a trait that is unique to pathogenic fungi. In addition, from our study we observed that the hyphae grow irregularly on the root surfaces. The hyphae of most endophytes deviated from the direction along the root axes when they grew inside cells (Abdellatif et al., 2009; Mebeaselassie et al., 2015b). So it is normal to see hyphae grew in irregular directions when they colonized living cells. In addition, to characterize *Arabidopsis* root invasion by *U. virens* at the molecular level, we used different well-established foliar defense genes. We have confirmed that *U. virens* was able to keep an intimate biotrophic relationship with the hosting *Arabidopsis* root cell for over at least 96 hpi without showing any significant apparent loss of the host’s cell viability. Such kind of tissue adapted fungal infection strategy was also previously reported by Marcel et al. (2010) on rice roots by *Magnaporthe oryzae*.

Generally, PR genes and proteins accumulate rapidly at the intra-or extra-cellular level under various biotic and abiotic stimuli, including fungal, elicitor and physical or chemical treatments (Van-Loon, 1985; Van-Kan et al., 1992; Van-Loon and Van-Strien, 1999; Graham et al., 2003). We demonstrate in this work that *A. thaliana* may serve as a good model host species to study the interaction between infected plants and the rice false smut fungus *U. virens.* Regardless the germination and growth of the *U. virens* fungal spores, our results show that *Arabidopsis* plants were able to activate different defense strategies in order to limit the damage and destruction which is caused by this biotrophic fungus. Within the first 24 h of post infection, we have observed plant defensin and pathogenesis related genes that are used for activating the plant defense system. These and other markers combined together and form a HR. The HR is known to play a very significant role in limiting or reducing the extent of damage which is caused by different pathogens (Dempsey et al., 1998).

Similar to our current observation, *PR-1* and *PDF1.2* expression was also induced in Col-0 upon infection with *M. oryzae* strains. *PR-1* expression was induced at 2 dpi but decreased at 3 dpi, however, unlike *PR-1*, the expression of *PDF1.2* was further increased at 3 dpi in Col-0 which is in congruence with the current finding (Park et al., 2009). Interestingly, the expression of both *PDF1.2* and *PR-1* genes decreases at 48 hpi and then increases at least threefold at 72 hpi and thereafter. This pattern of expression has been described when the plant needs to sense the pathogen and mount the resistance response, which in turn triggers a defense response (Govrin and Levine, 2000). Therefore, our results suggest that, although the rice false smut fungus isolates are able to infect and grow on *Arabidopsis*, this infection has set off a defense response within the plant. Similarly *U. virens* was able to colonize and infect *Arabidopsis* roots, flowers, and pods and it is witnessed by the increased levels of *PR1, PR2, PR5* as well as *PDF1.1* transcripts. Several *PR* proteins were identified so far that have been implicated in plant defense. Based on the initial speculation, defensin proteins are assumed to be present in large amounts in seeds and seedlings and are useful to give protection for the seeds and seedlings from soil-borne pathogens (Terras et al., 1995). However, unlike the previous speculation, defensins have much broader expression patterns and could be able to express in different plant organs (Kragh et al., 1995; Terras et al., 1995).

The use of plant models such as *Arabidopsis* is very instrumental in addressing the mechanisms of plant–microbe interactions since model plants are able to advance our knowledge in order to describe the plant immune system. In conclusion, here we reported a novel pathosystem based on *U. virens* and *Arabidopsis* and found that rice pathogenic *U. virens* transformed colonies were able to infect and colonize endophytically on the different parts of the *Arabidopsis* plant. Since the processes that determine the outcome of an interaction between a microbial pathogen and a host plant are complex, understanding the molecular details of these interactions, like the pathogen genes required for infection, effective host defense responses as well as mechanisms by which host and pathogen signaling networks are regulated, might be utilized to design new plant protection strategies. Generally, the established *A. thaliana–U. virens* pathosystem could be able to expand the model systems investigating fungi–plant interactions, and will facilitate a full understanding of *U. virens* biology and pathology.

## Author Contributions

MA and JL design experiment; MA conducted the experiment; MA analyzed the data; MA and JL wrote the paper.

## Conflict of Interest Statement

The authors declare that the research was conducted in the absence of any commercial or financial relationships that could be construed as a potential conflict of interest.
